# Fetal Alcohol Spectrum Disorder in a Newborn

**DOI:** 10.7759/cureus.28836

**Published:** 2022-09-06

**Authors:** Tejal Patel, Sameer Narula, Elena Naderzad, Daven Early, Thiagarajan Nandhagopal

**Affiliations:** 1 Medical School, Ross University School of Medicine, Miramar, USA; 2 Pediatrics, Kern Medical Center, Bakersfield, USA; 3 Internal Medicine, Ross University School of Medicine, Miramar, USA

**Keywords:** alcohol-related birth defects, alcohol-exposed pregnancy, fetal alcohol effects, teratogen, prenatal alcohol exposure, maternal alcohol use, fetal alcohol syndrome, fasd, fetal alcohol spectrum disorder

## Abstract

Fetal alcohol spectrum disorder (FASD) can be suspected in newborns based on the maternal history of alcohol use during pregnancy and compelling physical attributes present at birth. The infrequency with which it is assessed during infancy makes FASD evaluation in newborns a formidable diagnostic challenge.

Our objective was to evaluate a three-day-old male infant born at 37 weeks gestation via vaginal delivery to a mother with admitted excessive alcohol intake for FASD. On physical appearance, the newborn had evidence of smooth philtrum and thin vermillion border, which are two of the three cardinal facial characteristics for dysmorphology. We propose that early detection is necessary to improve the management of neurodevelopmental concerns such as behavioral issues or heart and vision problems.

## Introduction

Fetal alcohol spectrum disorders (FASDs) have been used to describe a group of conditions that affect people exposed to alcohol in utero. These include fetal alcohol syndrome, alcohol-related neurodevelopmental disorder, alcohol-related birth defects, and neurobehavioral disorder associated with prenatal alcohol exposure. FASDs are typically related to one another with different diagnoses presenting later in life.

FASDs are generally suspected in childhood based on a prenatal history of maternal alcohol use disorder and the presence of fundamental physical attributes for FASD [1,2]. In 2016, the clinical guidelines to diagnose FASD were consolidated and updated to include neuropsychiatric evaluation, the presence of behavioral changes, or developmental delay [3].

We propose that a newborn can be diagnosed with FASD using a new algorithm based on the current guidelines. The first step would be to confirm prenatal exposure to alcohol. Next, two of the three cardinal facial features of the smooth philtrum, thin vermillion border of the upper lip, and short palpebral fissures must be seen. Lastly, the use of the Revised Dysmorphology Scoring System (RDSS) presented in the Updated Clinical Guidelines for FASD to determine whether or not the patient is a candidate for a condition that is not yet determined under FASD. The RDSS grades are based on the presence and/or severity of features commonly seen in FASD. The current guidelines indicate a score of 5 or greater can be indicative of FASD. However, because developmental, behavioral, psychiatric, or some neurological delays are generally unable to be determined in a newborn, expanding the diagnostic criteria is not an unreasonable decision [3].

The importance of diagnosing FASD early on in a newborn can have several beneficial effects. The patient can be monitored for developmental delay or behavioral and psychiatric issues to prevent the worsening of signs and symptoms [4]. It can also be beneficial to ensure the child is being watched and periodically evaluated for warning signs of speech, gait, cardiac, and vision complications of FASD [1,2]. In this article, we discuss this in further detail.

## Case presentation

The patient, referred to as “John,” presented as a three-day-old male infant. He was born at an estimated 36 weeks gestational age via spontaneous vaginal delivery.

His APGAR scores were 9 and 9, his birth weight was 2,750 g, and his head circumference was 31 cm. At three days old, he was 2,625 g (lost 4.5% of his birth weight). Physical examination was significant for smooth philtrum and thin upper vermilion border which raised suspicion for FASD. Additionally, labs were suggestive of polycythemia.

The method we used to diagnose FASD involved three steps. The first was to evaluate the mother for maternal alcohol use disorder. Our patient’s birth mother, referred to as “Ms. Smith,” provided us with the necessary history. Ms. Smith was a 29-year-old G11P4 mother with a significant medical history of insufficient prenatal care, history of drug dependency and premature delivery, cholestasis of pregnancy, macrocytic anemia, and human papillomavirus infection. Based on an interview with Ms. Smith, she qualified for maternal alcohol use. Before pregnancy, Ms. Smith drank approximately 750 mL of vodka daily. During her pregnancy, she continued to drink the same amount for the first four months because she had not decided whether or not she wanted to continue the pregnancy. She tapered off alcohol for approximately three weeks once she decided to stay pregnant. However, she eventually became anxious about her pregnancy and financial concerns and began drinking a pint of vodka daily. She stated her last alcohol intake was the day prior to delivery.

The second step to diagnose FASD included the evident physical features seen on examination. It is generally accepted that after maternal alcohol use disorder is proven, you screen for positive dysmorphology facial evaluation for two out of three principal features, namely, smooth philtrum, thin vermillion border, and/or short palpebral fissures [3,5,6]. Our patient qualified for two of the three, namely, the smooth philtrum and thin vermillion border.

As is widely accepted in the 2016 Updated Clinical Guidelines for FASD, the next step is to conduct a neuropsychology evaluation [3]. However, because our infant was three days old, this evaluation was not possible. Hence, our third step instead was to use the RDSS to compare our patient to the scoring system, as shown in Table 1.

**Table 1:** Revised Dysmorphology Scoring System with our patient John’s data. Per the Updated Clinical Guidelines for Diagnosing Fetal Alcohol Spectrum Disorderspublished* *in 2016 [3]. FASD: fetal alcohol spectrum disorder; OFC: occipitofrontal circumference; palpebral fissure length; IPD: interpupillary distance; ICD: inner canthal distance; CHD: congenital heart disease

Features of FASD	Possible score	Subject’s score	Subject’s feature
OFC <10%	3	3	Yes
Growth deficiency			
Height <10%	2	0	No
Weight <10%	1	0	No
Short PFL (<10%)	3	0	No
Smooth philtrum	3	3	Yes
Thin vermilion	3	3	Yes
Hypoplastic midface	2	0	No
Epicanthal folds	2	0	No
Decreased IPD/ICD (<25%)	2	0	N/A
Flat nasal bridge	2	2	Yes
Altered palmar crease	2	0	No
Fifth finger clinodactyly	2	0	No
Long philtrum (>90%)	2	2	Yes
Anteverted nares	2	0	No
Camptodactyly	2	0	No
Ptosis	1	0	N/A
“Railroad track” ears	1	1	Yes
Heart murmur/confirmed CHD	0	0	No
Strabismus	1	0	N/A
Limited elbow supination	1	0	N/A
Hypoplastic nails	1	0	No
Prognathism	1	0	No
Hypertrichosis	1	1	Yes
Total possible score	41	15	

Our patient’s head circumference (OFC) was 31 cm, below the 10th percentile of 33.01 cm. The normal palpebral fissure length (PFL) of a newborn is 1.79 ± 0.17 cm, but our patient’s palpebral fissure was 1.8 cm. We also noted a smooth and long philtrum, greater than the 90th percentile. Additionally, our patient also had a flat nasal bridge, railroad track ears, and hypertrichosis.

**Figure 1:**
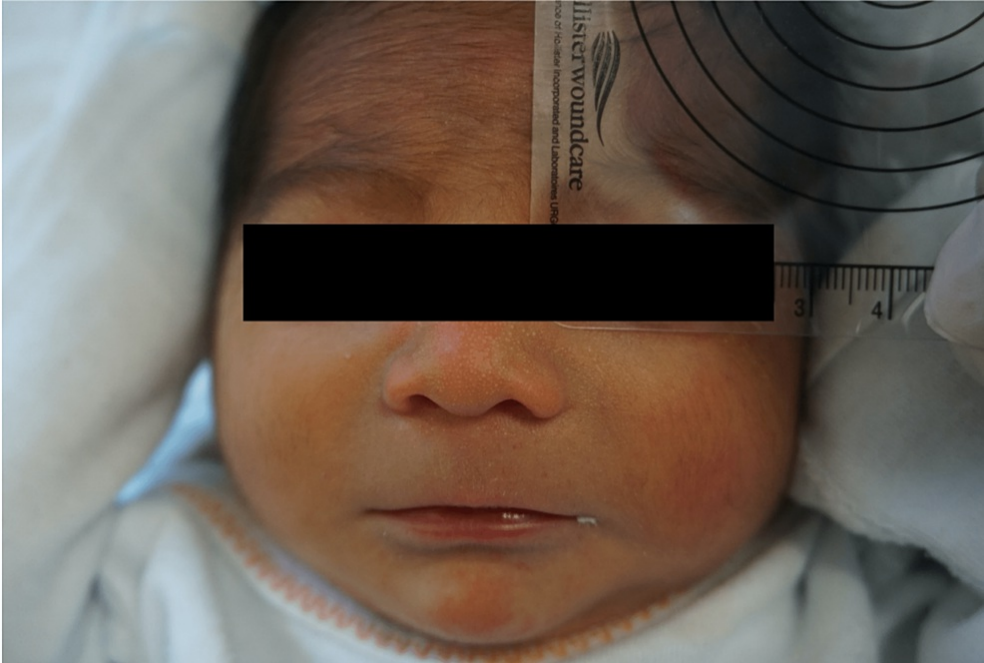
Cardinal facial features of dysmorphology. Smooth philtrum, thin vermillion border of the upper lip, mild hypertrichosis, and a flat nasal bridge are evidenced here in John.

**Figure 2:**
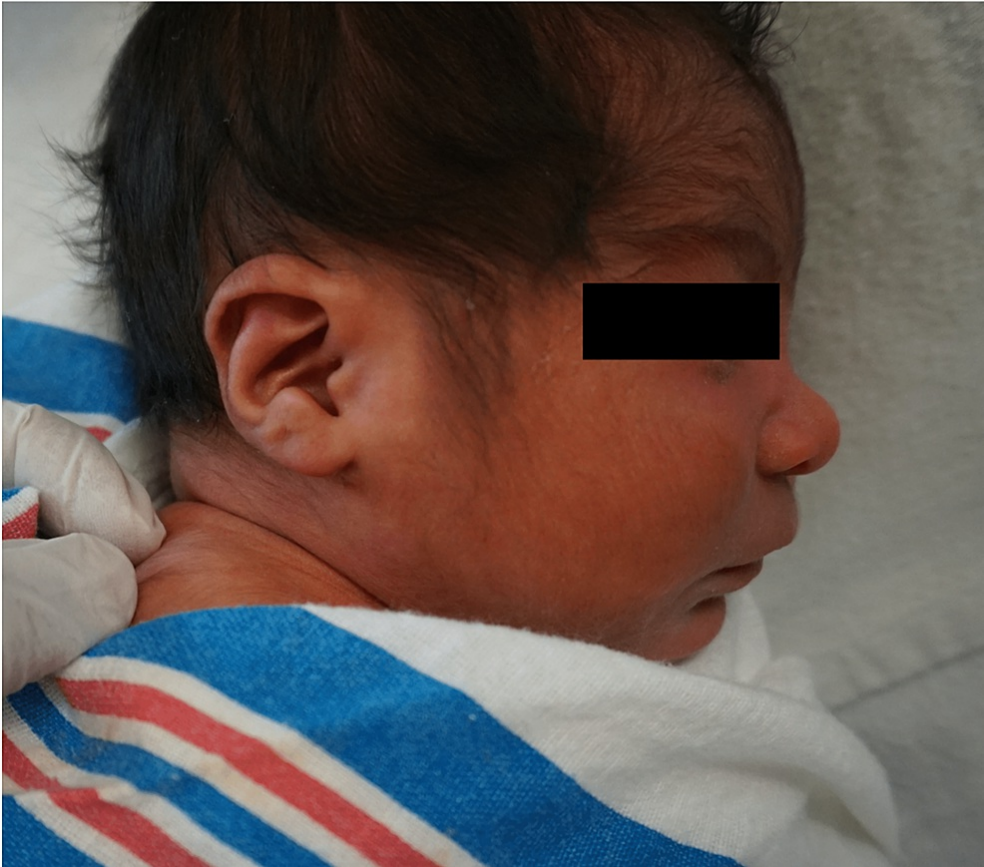
Additional physical attributes of FASDs. FASDs are also characterized by railroad track ears, a flat nasal bridge, and hypertrichosis. FASD: fetal alcohol spectrum disorder

Our results added to a score of 15. It has been established that a score greater than 5 is consistent with FASD. Figure 1 and Figure 2 show the observed features of FASD in the newborn, namely, smooth philtrum, thin vermillion, railroad track ears, flat nasal bridge, and hypertrichosis.

The patient was discharged with follow-up instructions; however, the patient was lost to follow-up before we were able to obtain the evaluation from his neurologist and assess for any developmental or neurological concerns.

## Discussion

There is no cure or specific treatment for FASDs, and the mental deficiencies persist throughout the lifetime of the affected individual. However, proper identification and early treatment can help mitigate these secondary neurodevelopmental symptoms. The physical symptoms, or the alcohol-related birth defects, of FASD have no cure. They are developed in utero and are permanent. If FASD is suspected, the pediatrician may refer the child to a neurologist, a developmental pediatrician, or another expert in fetal alcohol syndrome for evaluation and treatment [2,5].

Diagnosing FASD in patients requires a thorough assessment. Drinking levels throughout the initial development can be key to identifying different levels of neurological impairment. Although the physician should assess for cognitive abilities and learning and language difficulties as they would normally at any well-child visit, closer attention should be paid to those who may be affected to identify any delays for therapy immediately. The earlier these symptoms are found, the better the outcomes will be with specialized treatment. Early intervention services are available to help children from birth to three years of age learn important skills. Services include therapy to help the child talk, walk, and interact with others [1,2].

Intervention services may reduce some of the secondary disabilities and mental and neurological issues that a patient may encounter. These may include a special education teacher, a speech therapist, a physical and occupational therapist, and a psychologist. These services can help with social skills, walking, talking, and other delays the child may be displaying. Additionally, in the school systems, there may be special services that can help with learning and behavior issues.

Children with FASD have been shown to also display symptoms very similar to attention-deficit hyperactivity disorder (ADHD) and may meet the same diagnostic criteria in the Diagnostic and Statistical Manual of Mental Disorders, Fifth Edition. An approach to this is to treat these patients with the same medications to deal with the symptoms of attention and hyperactivity. These medications include stimulants which have been successful in treating ADHD. However, mixed results have been found with stimulant treatment in clinical studies on FASD. Non-stimulant medications such as atomoxetine are currently under clinical trial for the treatment of nerve developmental symptoms [4].

If the patient has health issues such as vision problems or heart abnormalities, surgical intervention or appropriate pharmacological treatment may be considered. About 25-50% of children with FASD suffer from strabismus, which can be corrected with patching of the normal eye to correct the deviation if identified early. Other problems may include cataracts, which can be corrected surgically, and nystagmus. The cardiac abnormality seen in FASD is most typically a ventricular septal defect. Depending on the severity of the defect, it may be asymptomatic; however, surgical intervention may be required in cases of cyanosis or Eisenmenger syndrome [1,6].

It is important to identify many of these symptoms or developmental delays as early as possible. Unfortunately, FASDs can often be missed or misdiagnosed due to underreporting of maternal alcohol use, or lack of characteristic facial traits leading to a misdiagnosis of ADHD. Early detection can lead to the best results in minimizing the symptoms for long-term development. In our patient, due to physical manifestations, and a thorough history from the mother, we were able to diagnose this patient early on. The mother had been closely communicating with one of our researchers to provide her with updates from various specialists she is working with before she was lost to follow-up.

## Conclusions

Based on maternal alcohol use history and principal facial characteristics for dysmorphology, we suggest that it is acceptable to diagnose a newborn with FASD using RDSS in lieu of a neuropsychology evaluation. We conclude that early detection of common issues such as learning and language difficulties can improve the child’s quality of life. Interventional services are available for children to develop important social and educational skills. With the assistance of various providers involved in child development, such as special education teachers and speech physical and occupational therapists, children can learn social, language, physical, and educational skills to prevent or mitigate delays in development. Additionally, medications can be prescribed by a psychiatrist for different symptoms such as distractibility, similar to ADHD. Visual symptoms, such as strabismus, can be corrected, and treatment may include eyeglasses, prisms, vision therapy, or eye muscle repair surgery. Cardiovascular pathologies can also be detected and observed in asymptomatic patients, and surgical intervention is an option for symptomatic patients.

Although there is no guideline to reverse FASD, we suggest that early identification and intervention can slow progression and manage secondary neurodevelopmental symptoms.
